# Network analysis of dairy cattle movement and associations with bovine tuberculosis spread and control in emerging dairy belts of Ethiopia

**DOI:** 10.1186/s12917-019-1962-1

**Published:** 2019-07-26

**Authors:** Getnet Abie Mekonnen, Gobena Ameni, James L. N. Wood, The ETHICOBOTS consortium, Stefan Berg, Andrew J. K. Conlan

**Affiliations:** 1 National Animal Health Diagnostic and Investigation Center, P. O. Box 04, Sebeta, Ethiopia; 2 Aklilu Lemma Institute of Pathobiology, Addis Ababa University, P. O. Box 1176, Addis Ababa, Ethiopia; 3 Disease Dynamics Unit, Department of Veterinary Medicine, University of Cambridge, Madingley Road, Cambridge, CB3 0ES UK; 4 Bacteriology Department, Animal and Plant Health Agency, Surrey, KT15 3NB UK

**Keywords:** Bovine tuberculosis transmission, Contact network analysis, Ethiopia, Scale free, Small world

## Abstract

**Background:**

Dairy cattle movement could be a major risk factor for the spread of bovine tuberculosis (BTB) in emerging dairy belts of Ethiopia. Dairy cattle may be moved between farms over long distances, and hence understanding the route and frequency of the movements is essential to establish the pattern of spread of BTB between farms, which could ultimately help to inform policy makers to design cost effective control strategies. The objective of this study was, therefore, to investigate the network structure of dairy cattle movement and its influence on the transmission and prevalence of BTB in three emerging areas among the Ethiopian dairy belts, namely the cities of Hawassa, Gondar and Mekelle.

**Methods:**

A questionnaire survey was conducted in 278 farms to collect data on the pattern of dairy cattle movement for the last 5 years (September 2013 to August 2018). Visualization of the network structure and analysis of the relationship between the network patterns and the prevalence of BTB in these regions were made using social network analysis.

**Results:**

The cattle movement network structure display both scale free and small world properties implying local clustering with fewer farms being highly connected, at higher risk of infection, with the potential to act as super spreaders of BTB if infected. Farms having a history of cattle movements onto the herds were more likely to be affected by BTB (OR: 2.2) compared to farms not having a link history. Euclidean distance between farms and the batch size of animals moved on were positively correlated with prevalence of BTB. On the other hand, farms having one or more outgoing cattle showed a decrease on the likelihood of BTB infection (OR = 0.57) compared to farms which maintained their cattle.

**Conclusion:**

This study showed that the patterns of cattle movement and size of animal moved between farms contributed to the potential for BTB transmission. The few farms with the bulk of transmission potential could be efficiently targeted by control measures aimed at reducing the spread of BTB. The network structure described can also provide the starting point to build and estimate dynamic transmission models for BTB, and other infectious diseases.

**Electronic supplementary material:**

The online version of this article (10.1186/s12917-019-1962-1) contains supplementary material, which is available to authorized users.

## Background

Ethiopia has huge livestock resources including cattle population of 60.4 million [1]. Cattle are the dominant species constituting 70–90% of the Ethiopian livestock producing households, and accounting for about 72% of the meat and 77% of the milk produced annually in the country, indicating its overriding role in generating smallholders’ income and in meeting domestic meat and milk consumption requirements [2]. At present, about 98% of the Ethiopian dairy cattle are of the Zebu breed and managed under extensive farming in agro-pastoral and pastoral systems. However, rapid urbanization is placing challenges to meet the demand for food (including dairy products) from an increasing population. The milk production potential of Zebu cattle is poor and as a result the possibility of meeting the increasing demand for milk and its products using the Zebu breed is minimal. Due to this situation, the Ethiopian Government, in its economic development strategy, has prioritized improvement of the breed of dairy cattle, pasture development and intervention on animal health to cope with the increased demand of milk and other livestock products [2]. The breed improvement plan focuses on breeding crosses of Holstein Friesian (HF) (*Bos taurus*) and Zebu (*Bos indicus*) breeds mainly by using artificial insemination services through synchronization wherever possible. Animals produced through cross breeding will have an added advantage of resilience to harsher environments in addition to increased milk production and dairy cattle productivity. Thus, the dairy development is of paramount importance particularly for the provision of employment opportunities (especially for women), poverty alleviation, and improvement of human nutrition and health [3]. As a consequence of these development efforts, intensive dairy farms and smallholder farms raising HF crosses are increasing in and around major urban centers.

The dairy farming is relatively well-developed in central parts of the country although it has also become an emerging sector in the peripheral regions [4]. The demand for the improved breed of dairy cattle for stocking of the emerging and/ or expanding farms in the peripheral regions is met mainly by the purchase of cross bred dairy cattle from the central areas of the country. However, the central part of the country has a high prevalence of bovine tuberculosis (BTB) [5–9]. The centrifugal trade of dairy cattle from areas with higher prevalence of BTB to areas with lower prevalence poses a high risk of transmission into the peripheral areas where much lower disease prevalence have been recorded in the diary sector [10]. Prevailing conditions such as developing infrastructures, national development plans etc., favor trade of cattle from long distances. Nevertheless, it has been well documented that animal movement within and between animal populations is a central driver of disease spread as pathogens can be transmitted over long distances via movement of infectious animals [11–17]. Understanding the structure of cattle movement networks and exploring the trade routes, volumes and frequency of dairy cattle movement in the Ethiopian conditions can inform how BTB and other infectious disease could potentially spread in the country. Studies on the impact of cattle movement networks and the associated risk of BTB transmission are lacking in Ethiopia. In the United Kingdom, movement of dairy cattle was estimated to be responsible for up to 84% incidence rate of BTB in herds [18]. In recent years social network analysis has become a tool of choice to link movement networks with transmission and dynamics of infectious diseases [19–24]. Although the application of social network analysis for studying disease transmission has not been common in developing countries, several studies have been conducted in Europe including the network analyses of the initial phase of the 2001 foot and mouth disease epidemic in the UK [12], the transmission of infectious disease in sheep population in the Scotland [16] and the spread of BTB and its control in the UK [18]. The main challenge in developing countries including Ethiopia, though suggested for more informed disease control [25], is a lack of animal identification, registration and traceability system in which data regarding cattle movement is recorded. While data scarcity and quality issues remain a problem, possible efforts to better understand the existing conditions need attention. Therefore, the purpose of this study was to understand the network structure using available cattle movement information, identify relevant network properties and explore associations with the epidemiology of BTB.

## Results

### Centrality measures

**Fig. 1:**
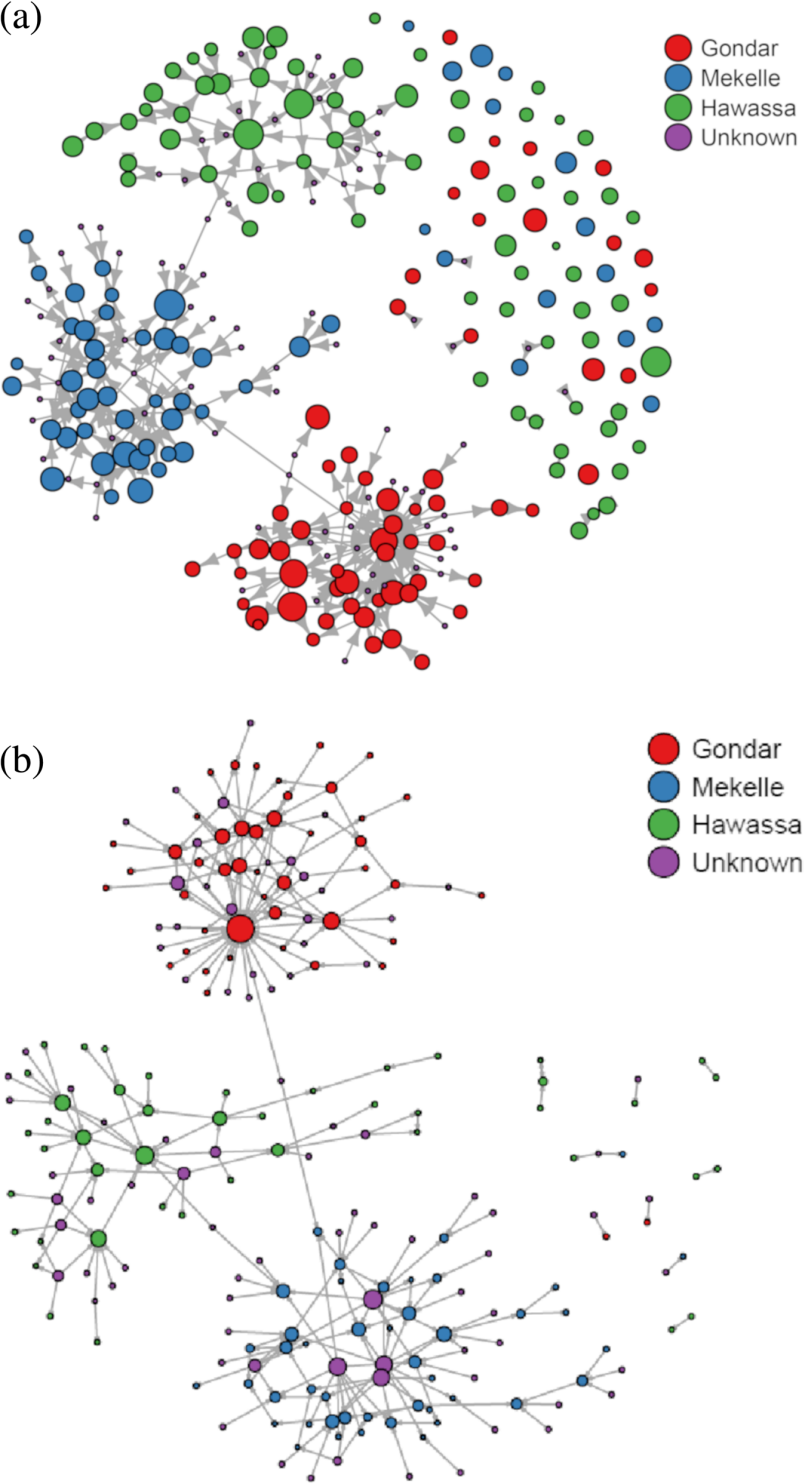
**a** and **b.** Network topology constructed based on dairy cattle movement data between September 2013 and August 2018; (**a)** vertex size based on herd size; (**b**) vertex size based on the number of connections; vertex colors indicated regions/sites, arrows indicate direction of animal movement

Analysis of the established network due to dairy cattle movement within the study sites identified 278 farms/sites as nodes and 584 connections (cattle movement records dated between September 2013 and August 2018) as edges. The cattle movement network topology for the full network is presented in Fig. 1a & b. Among farms 81% (225/278) had at least one connection with any of the farms, majority of which (68%, 190/278) had connections lower than five compared to farms having at least five connections (13%, 35/278) accounting, respectively, for 55.5% (324/584) and 45.5% (260/584) of the overall connections in the network. However, 19% (53/278) of the farms in the network did not have any connections with regards to dairy cattle movement (Additional file 1: Table S1).

The outputs of node centrality measures are presented in Table 1. Each farm was observed to have a median link of 1 (range: 0 to 37) with other farms, as measured by the degree centrality. This was found to be consistent across all sites. The outdegree centrality for any of the node in the full network was also observed to show a median of 1 while the indegree showed a median of 0 but fewer farms had higher number of incoming connection (range: 0 to 29). These centralities in the full network were found to correlate negatively (Spearman correlation, r = − 0.25). Higher level of farm centrality due to closeness was observed in the full network indicating requirement of only very fewer steps (average 0.01) to access every other farm from a given farm in the network. In this regard, Gondar showed higher level of farm centrality compared to other sub-networks. Fewer farms were observed to show a higher betweenness of up to 299 connections although majority of them showed very little or no potential as explained by the median value of betweenness centrality. The probability of well-connected farms in the full network to connect with other well-connected farms was observed to be lower (Eigenvector of about 3%) compared to sub-networks specific to the study sites.

**Table 1:** Node centrality metrics of cattle movement network (median values for degree, indegree, outdegree and betweenness; mean for closeness and eigenvector)

Centrality^a^	Centrality values (ranges)			
	Mekelle	Gondar	Hawassa	Full network
Degree	1 (0, 11)	1 (0, 37)	1 (0, 12)	1 (0, 37)
Indegree	0 (0, 11)	0 (0, 29)	0 (0, 6)	0 (0, 29)
Outdegree	0 (0, 5)	1 (0, 8)	1 (0, 12)	1 (0, 12)
Closeness	0.019 (0.01, 0.02)	0.0004 (0.0001, 0.0005)	0.04 (0.01, 0.05)	0.01 (0.004, 0.013)
Betweenness	0 (0, 15)	0 (0, 299)	0 (0, 30)	0 (0, 299)
Eigenvector	0.11 (0, 1)	0.1 (0, 1)	0.15 (0, 1)	0.03 (0, 1)

^a^Metrics were calculated separately for each study sites and for the full network

### Network properties

Results of the dairy cattle movement network analysis based on selected network parameters are presented in Table 2.

**Table 2:** Network metrics of cattle movement network calculated separately for each site and then for the full network

Parameter	Metrics values			
	Mekelle	Gondar	Hawassa	Full network
Diameter	2	5	6	6
Average shortest path length	1.36	2.1	2.1	1.96
Density	0.01	0.013	0.012	0.004
Reciprocity	0.027	0.018	0	0.014
Assortativity (based on degree)	-0.04	-0.32	-0.01	-0.17
Global clustering coefficient (CC)	0.15	0.18	0.07	0.13
Modularity^a^	0.68	0.49	0.60	0.72
Components (GWCC)	33	18	15	63
Community (based on greedy optimization)	38	24	22	73
Centralization (by degree)	0.05	0.19	0.05	0.06

^a^Modularity value near to 0 indicates that the network considered is close to a random one (barring fluctuations), while a value near to 1 indicates strong community structure

The full cattle movement network displayed lower density of connections, which means that only 0.4% of the possible links were present, suggesting a very lower overall cohesiveness of the network and illustrating the local/ regional nature of trade in Ethiopia. A minimum of six steps were required for connecting the two most distant reachable farms in the network, as measured by the network diameter. Visualization of the path of the network diameter showed that it began from farm ID 9F011 and ends at farm ID 9F003, all the farms along the path being located in Hawassa only. In the full network, the assortativity measurements based on degree centrality showed that farms with higher degree centralities tend to preferentially connect with farms of lower degree centrality measures, and the tendency was found to be stronger for Gondar (− 0.32) as compared to that of either Mekelle (− 0.04) or Hawassa (− 0.01). Network centralization based on degree centrality demonstrated that the sub-network in Gondar was more centralized although the overall network showed more of decentralized tendency (Table 2).

**Fig. 2:**
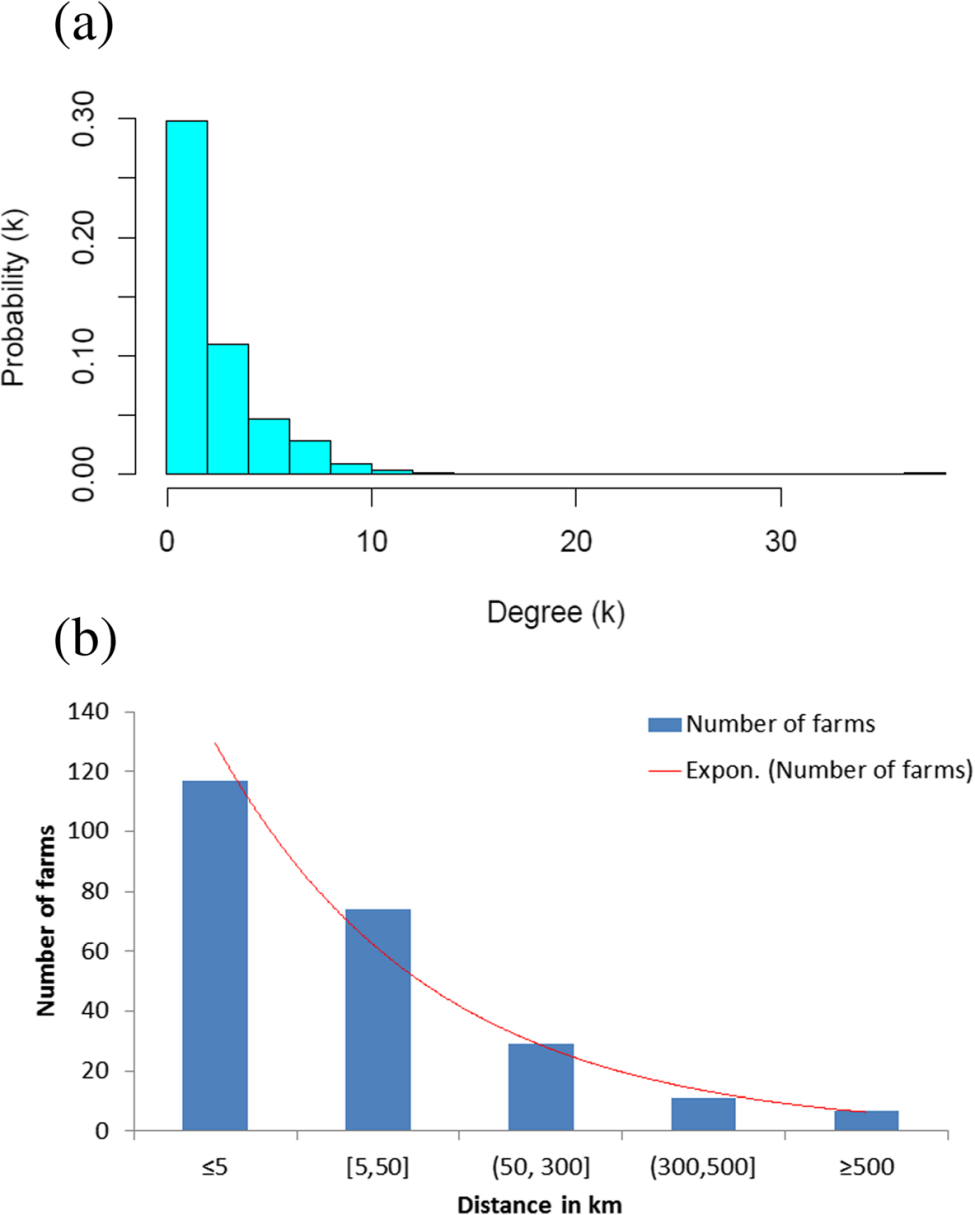
**a** and **b** Degree and Euclidian distance distributions of dairy cattle movement network. (**a**) Degree distribution; (**b**) Distribution of Euclidian distance among farms

The average of the local clustering coefficient of each farm (called the global clustering coefficient) for the full cattle movement network was 0.13 (Table 2). When comparing the sub-networks, the one in Gondar was more clustered while the one in Hawassa was less clustered than the sub-network in Mekelle. The average shortest path length for the full network was 1.96 which means very few steps could be required for a farm to access other farms in the network. To ascertain whether the full network displayed a small world structure, the values of average shortest path length and clustering coefficient were compared with that of the random network [26]. Accordingly, the random network showed a much lower clustering coefficient (0.01, about 13 times lower) and higher average shortest path length (8.5) proving that the established cattle movement network was highly clustered and efficient to reach out quite easily, showing that the real network displayed a small world structure. Considering the geographic Euclidean distance between the source and end farms (range: 0.02–709 km), 49% of the distances among farms were below 5 km, and only about 8% of the distances were greater than 300 km, showing that cattle movement in most instances were localized and it was only in few cases that cattle were moved from distant places (Fig. 2b).

The degree distribution of the farms in the dairy cattle movement network was not normally distributed. It was skewed to the right indicating that only very fewer farms were highly connected compared to the majority of the farms (Fig. 2a). The distribution is well described by a power-law distribution at alpha and R^2^ of 1.62 and 0.86, respectively. As a consequence of this large heterogeneity in the number of connections per farm, the existence of hubs (farms with high outdegree) and authorities (farms with high indegree), we conclude that the cattle network demonstrates a scale free structure.

### Key actor analysis

Farms playing a critical role in the cohesiveness of the network were identified based on the correlation analysis of node centrality measures (Additional file 2: Table S2). The overall correlations among node centralities were low to high. Higher correlation (r = 0.83) was observed between closeness and eigenvector centralities, while weaker correlation (r = 0.24) was observed between eigenvector and betweenness centralities and thus applied to detect critical farms in the network. Accordingly, three dairy farms with farm ID’s 7F020 from Gondar, 9A038 from Hawassa, and 8F007 from Mekelle, were identified as critical, serving both pulse taker’s and gate keeper’s roles within their respective sub-networks. The identified critical farms were considered as the nucleus for the structural functionality of the sub-networks, in fact they were essential in connecting part of the sub-networks that would otherwise be isolated. A couple of farms were also recognized to serve as either pulse taker’s or gate keeper’s role in Hawassa and Mekelle; however, no farm was observed to function either of the roles in Gondar. In the full network, farm ID 7F020 (from Gondar) served both the attributes of pulse taker’s and gate keeper’s function but none of the remaining farms showed no role for the functionality of the full network (Additional file 4: Figure S1).

### Cohesive analysis

The dairy cattle movement network was organized in 4 core sub-groups of k: 3, 2, 1 and 0 with size of 9, 89, 126 and 53 nodes, respectively (Additional file 5: Figure S2). Among the farms involved in the network, there were 63 GWCC, 53 of which contained only one node, the remaining components contained between 2 and 204 nodes. However, the network has no giant strong connected component. A measure of the quality of community structure in the dairy cattle movement network was determined in terms of the modularity, estimated at 0.72 (Table 2), indicating higher tendency of intra-community connections than the same community structure would present if the connections would be rewired under random network. Community detection based on greedy optimization algorism identified 73 communities within the connected network. The largest community involved 46 farms while the smallest had one farm. Three of the top largest communities contained 119 farms, accounting for 43% of the farms in the network, while the remaining 57% of the communities had between 1 and 20 farms per community. Distributions of communities in majority of the cases were restricted to the study sites but there were crossing of few communities between regions/sites. Fewer farms in Gondar and Mekelle had connections with farms in Hawassa and thus communities involving such farms were observed to cross over. Few other smaller communities in Mekelle and Gondar were also observed to cross each other although there were no connected farms in between (Additional file 6: Figure S3).

### Network reliability

**Fig. 3:**
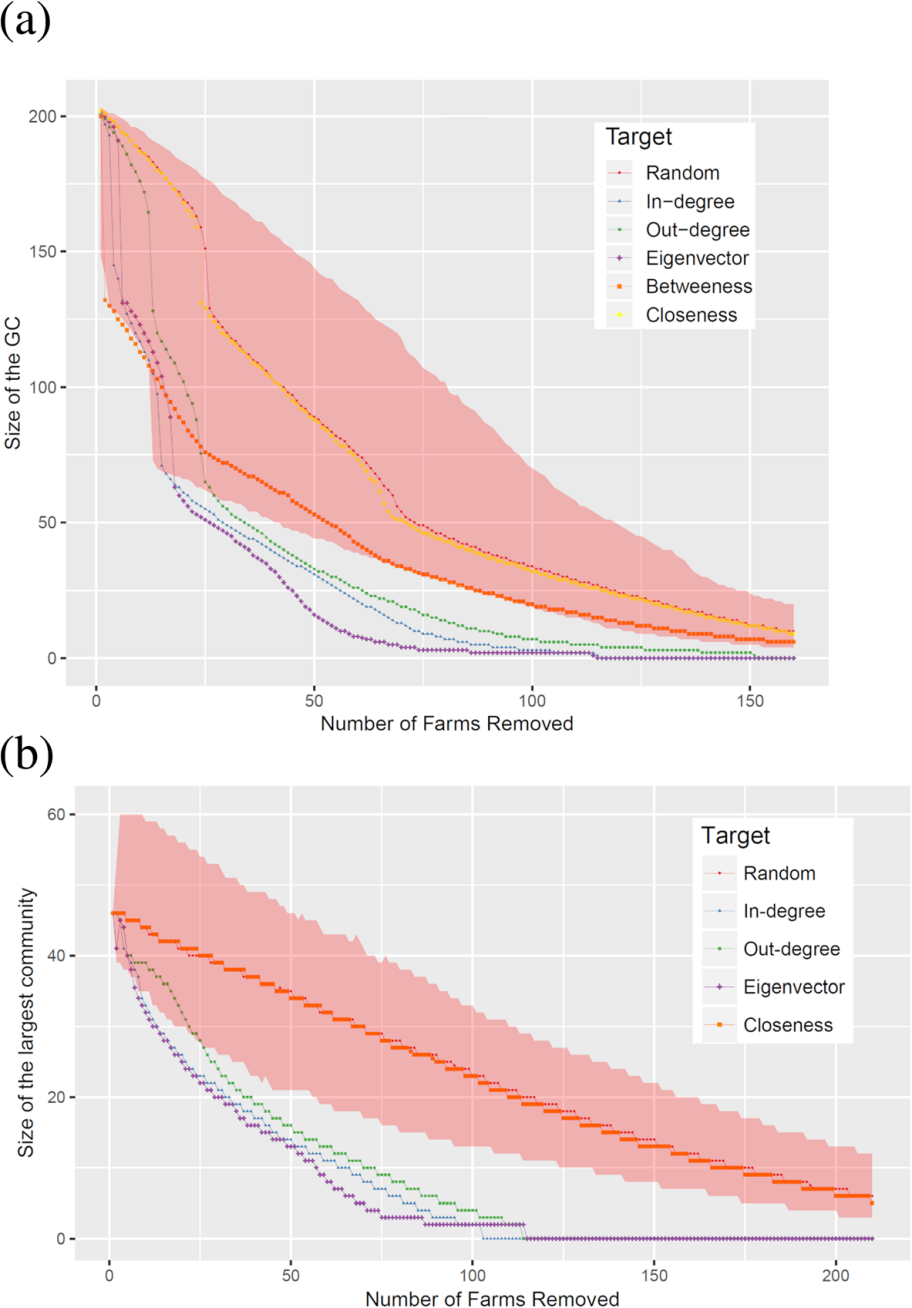
**a** and **b.** The effect of targeted farm removal, driven by the different centrality measures on the fragmentation of the GWCC (**a**) and largest community size (**b**) of the cattle movement network. The y axis shows the size of the GWCC (**a**) and the largest community size (**b**), and the x axis shows the number of farms removed from the network. The graph was based on the median of the centrality measures after 1000 simulations

A percolation analysis was carried out to assess the vulnerability of the cohesion of the network structure as measured by the size of GWCC and largest community. Figure 3a and b compare the impact of selective removal of farms according to their centrality measures to random selection.

Targeted removal of farms in the network based on decreasing order of the betweenness, indegree, outdegree and eigenvector values showed remarkably faster changes in the network structure with faster reduction on the size of GWCC compared to random removal (Fig. 3a). Removal of farms based on their betweenness is the first to fall outside the random targeting simulation envelope but then out performed by in-degree, out-degree and eigenvector centrality. Therefore it seems that the GWCC can be disintegrated if one use in the order of eigenvector, indegree, outdegree and betweenness centrality for the targeted removal of vertex compared to the random removal. Removal of about 24% (50/150) of the farms in the network could reduce the size of the GWCC by more than 85% (174/204). In contrast, removal using closeness centrality did not disintegrate the network structure better than random removal. The effect of targeted removal on the size of the largest community was also investigated. The largest community size in the network dropped promptly when farms were removed based on the value of their eigenvector centrality followed by the indegree and then the out-degree; however, removal based on the values of the closeness centrality showed a similar pattern of reduction with random removals (Fig. 3b).

### BTB infection and features of cattle movement network

The herd level prevalence of BTB was compared between farms which had at least one incoming link to those which had no any incoming connection. Accordingly, a 27% positivity to the tuberculin test was observed among the connected farms compared to 18% positivity among the non-connected ones. We used a logistic regression model to estimate the strength of association between network characteristics and BTB positivity. We were also interested in quantifying the effect of batch size of movement and Euclidean distance between herds. However, due to missingness in the data it was necessary to estimate a second model to explore these two additional factors. The response variable for both models was the probability of a herd having any positive animals (defined by presence of any reactor animals within herd) and predictor variables were selected based on a univariate screen with a *p* value *< 0.25*.

Results of the regression model with network characteristics as predictor variables are shown in Table 3. Within the network, some farms were observed to have higher level of throughput as demonstrated by higher values of their indegree and outdegree measures. The regression model estimates that the log odds of ‘farm BTB positivity’ increased by 120% with a unit increase of the indegree (adjusted OR 2.2). On the other hand, a decrease on the likelihood of BTB positivity by 43% (adjusted OR = 0.57) was observed among farms that had one or more outgoing animals (outdegree ≥1) compared to farms that maintained their animals (outdegree =0). Comparing the relative closeness between farms on the ‘farm BTB positivity’, farms having closeness centrality value of higher than average showed a decrease by 60% on the odds of ‘farm BTB positivity’ (adjusted OR 0.4). On the other hand, farms having eigenvector centrality of at least the average value showed significantly (*p* < 0.05) higher likelihood of being BTB positive (adjusted OR 3.3).

**Table 3:** Point estimates of node characteristics by logistic regression univariate and multivariable models for herd level BTB positivity (*n* = 252)

Risk factors	Class	Univariate		Multivariable	
		Crude OR (95% CI)	*P*	Adjusted OR (95% CI)	*P*
Availability of incoming connection (indegree)	No	–	–	–	–
	Yes	1.72 (0.9, 3.2)	0.077	2.2 (1, 5)	0.054
Availability of outgoing connection (outdegree)	No				
	Yes	0.39 (0.2, 0.72)	0.003	0.57 (0.3, 1.2)	0.170
Connecting other neighboring farms (betweenness)	No	–	–	–	–
	Yes	1.5 (0.7, 3.2)	0.304	–	–
Closeness	< average	–	–	–	–
	≥average	0.55 (0.3, 1)	0.067	0.4 (0.2, 1)	0.058
Eigenvector	< average	–	–	–	–
	≥average	2.2 (0.84, 5.5)	0.093	3.3 (1.2, 9)	0.022

The second regression model, constructed to estimate the herd BTB positivity using batch size and Euclidean distance as predictor variables, suggested that a batch size of ≥2 cattle could significantly increase the BTB positivity of a farm compared to farms with a batch size of one or no incoming cattle (Table 4). Euclidean distances between the source and destination farms were also found to be associated with BTB positivity of farms. Farms that had cattle sourced from distant farms /sites were more likely to have BTB infection compared to farms that had cattle sourced from closer farms/sites.

**Table 4:** Point estimates of risk factors by logistic regression univariate and multivariable models for herd level BTB positivity (*n* = 181)

Risk factors	Class	Univariate		Multivariable	
		Crude OR (95% CI)	P	Adjusted OR (95% CI)	P
Euclidean distance in km		1.009 (1.005, 1.01)	< 0.001	1.007 (1, 1.01)	0.002
Batch size (number of animals)	≤1	–	–	–	–
	2–4	2.6 (1.2, 5.7)	< 0.001	2.7 (1.3, 6)	0.012
	≥5	14.6 (7.2, 31)	< 0.001	12 (5.8, 26.5)	< 0.001

The herd and animal level BTB positivity were also evaluated across the community structure in the network. The number of infected farms in the community were moderately correlated with community size (Spearman correlation r = 0.63); while, proportion of infected animals was observed to fairly correlate (Spearman correlation r = 0.45) with community size. To test the effect of community structures on BTB positivity, we constructed univariate logistic regression models for the animal and herd level BTB positivity independently. The response variable was either the herd positivity or animal positivity, and community size was predictor variable in both cases. Accordingly, the univariate regression analysis at herd level demonstrated that a unit increase on the community size significantly increased (*p* < 0.05) the log odds of ‘farm BTB positivity’ by 0.23 units (crude OR = 1.3, 95% CI: 1 to 1.6). Similarly, the regression on animal level, showed an increase on the log odds by 0.25 units (crude OR 1.3, 95% CI: 1.1 to 1.9) due to a unit increase on the community size (Additional file 7: Figure S4).

## Discussion

In the present study, the dairy cattle movement network and its impact on the spread of BTB were investigated in three emerging dairy belts of Ethiopia, namely the cities of Hawassa, Gondar, and Mekelle using a social network analysis in conjunction with tuberculin testing. The result of this study showed a higher prevalence of BTB in farms that had a link history within the network than in farms that had no connection in the network suggesting that the possibility of BTB transmission through the movement of the dairy cattle. This observation is substantiated by earlier studies that indicated the role of animal movement in the spread infectious diseases [18, 19, 23, 27].

Higher variation in the number of connections per farm and betweenness in the network structure illustrated the heterogeneity of the number of connections per farm. Highly connected farms, which can be called hubs, may serve as super spreader of BTB once infected. If a farm serving as hub is removed from the network, spread of infections might be reduced with better effect than removal of other farms with lower degree and betweenness in the network [28]. Farms with higher indegree tend to have lower outdegree suggesting the absence of farms which are both likely to become infected and to transmit infection playing an important role in facilitating BTB transmission within the network [27]. In farms with incoming connectivity, an increased odds of BTB positivity was observed (adjusted OR = 2.2, 95% CI: 1 to 5); on the other hand, farms having outgoing connections showed a decrease of odds ratio by 43% (adjusted OR = 0.57, 95% CI: 0.3 to 1.2) compared to farms that had no any connection. The increase due to incoming connectivity could be due to the purchase of infected cattle from farms which did not know the BTB status of the animal and thus the buyer took the risk by chance, or rarely, fewer infected farms might sell reactor animals hiding the BTB status instead of culling since there is no policy in the country enforcing them not to do so. This can further be corroborated by supplemental data that cattle sourced from other farms/sites showed significantly higher level of BTB infection than the preexisting ones [29]*.* Whereas, the decrease of positivity on farms with outgoing connections showed the impact of prompt removal of reactor animals from the herd; and repeated skin testing and removal of reactors could help to create apparently BTB clean farm [30, 31]. In addition to the links, batch size has also been evidenced to relate with BTB infection of a farm. This study found that farms introducing a batch size of ≥2 cattle showed an increased likelihood of BTB positivity compared to farms which introduced one animal or not introducing at all. This is in line with previous suggestion that restricting the number of traded dairy cattle could prevent BTB transmission [32]. Although nearly half of the moved cattle were sourced from within 5 km distances (Fig. 2b), cattle sourced from distant origins showed more likelihood of BTB infection suggesting the risk of BTB spread through cattle traded/moved from far areas. This was in line with the trend of dairy cattle movement following the government’s dairy expansion plan where cattle moved from the central parts of the country where the dairy sector was well developed but with high BTB prevalence. This could also be substantiated to the socio-cultural reasons that BTB infected cattle, if not culled, are more likely to be traded to distant areas instead of closer or neighbor farms.

The present study suggests that the cattle movement network between Ethiopian dairy herds has small world properties due to its higher global clustering coefficient (CC = 0.13) and a relatively short average path length (only 1.96 steps) compared to a random network generated with same number of nodes and connections. The global clustering coefficient for the present network is lower showing that the local farm to farm interactions are at smaller level and thus spread of BTB among themselves are inconsistent [33] and the spread may be relatively slow [34]. Transmissions of infectious agents have been suggested to be quicker in networks with similar properties but with higher clustering coefficients [17, 35]. In small world networks, the BTB spread may cover most clustered farms relatively quickly; however, the presence of fewer long-range connections suggest the potential of disease breakouts in less clustered farms [36].

The right skewed degree distribution and its power-law fit of the cattle movement network also suggest a scale free property. Fewer farms having high number of connections with majority of the farms serving as hubs, are at greater risk in getting the BTB infection and once infected can be potential supper spreaders to many other farms connected to them [27]. Hubs can not only play a role as super spreaders but also as maintainers of BTB infection. Previous studies of infectious disease epidemics on scale free network demonstrated faster epidemics spread due to the presence of hubs [37–40].

Higher-order relationships between farms in the full network shows negative assortative mixing suggesting that highly connected farms tend to connect with less connected farms, and this relationship was found to be stronger in Gondar compared to that either in Hawassa or Mekelle, demonstrating the potential in accelerating BTB spread within their respective sub-networks [11]. Frequent connections between highly and less with well-connected farms have been substantiated to slow the spread of infectious disease as compared to networks with positive assortative relationships [27]. Negative assortative relationship as observed in our networks can be beneficial for BTB control, since implementing control measures such as movement restriction, culling and /or increased biosecurity measures to highly connected farms protects less connected farms attached to them [23].

Disintegration of giant connected components and biggest communities restricts the spread of infectious diseases [23]. Targeted removal of farms in the cattle movement network to fragment its cohesiveness can be considered as an effective strategy to identify farms playing vital role in disease transmission and impose effective disease control measures such as implementation of movement restriction, vaccination or diagnostic testing [24, 27, 41]. In this regard, the present data suggested that targeting on the top 5% of highly connected farms based on their eigenvector value would reduce the cohesiveness of the network by nearly 35% (as explained from the fragmentation of the GWCC), and if we increase the target to 15% of the connected farms then the cohesiveness would be reduced considerably (reduce by greater than 75%). Targeted removal based on the eigenvector value also showed good effect on fragmentation of the biggest community but this is at lower rate compared to the effect on GWCC as a measure of network resilience. Fragmenting the network cohesiveness relatively quickly suggests that the rate at which BTB spread among farms in the network could be restricted. Targeted removal of farms based on eigenvector values signifies that targeted interventions could be one possibility for disease control. However, the effectiveness may be progressive for BTB due to its chronic nature and take longer period to recognize the intervention impact compared to acute infections. Thus, the control efficacy can be enhanced if targeted intervention is combined with other control measures such as implementation of good biosecurity measures, movement restriction from BTB endemic areas, reduce the number of traded cattle, segregation and culling of skin test positive animals.

In this study, the information used for the network analysis was based on the recall of the respondents due to absence of recording system. However, possible verifications were made by involving other family members, animal attendants who had stayed at the farm for longer period and local extension workers who closely support the farming system. The analysis was made focusing mainly on cattle movement. Other possible pathogen transmission pathways such as movement of other species of animals, people and vehicles, neighborhood, sharing of bulls and facilities were not considered. Characterization of the cattle movement network is not an easy task especially in developing countries where there is no proper recording system.

## Conclusions

This paper provides a first estimate and quantitative description of the cattle movement patterns between dairy herds in Ethiopia and suggests that control interventions could be targeted to achieve a greater impact. Assessing the relative impact of alterative control strategies such as test-and-slaughter, vaccination and movement restrictions will require the development of dynamic transmission models. This data provides the starting point to build and estimate such models for BTB, and other infectious diseases, in Ethiopia.

## Methods

### Study sites

**Fig. 4:**
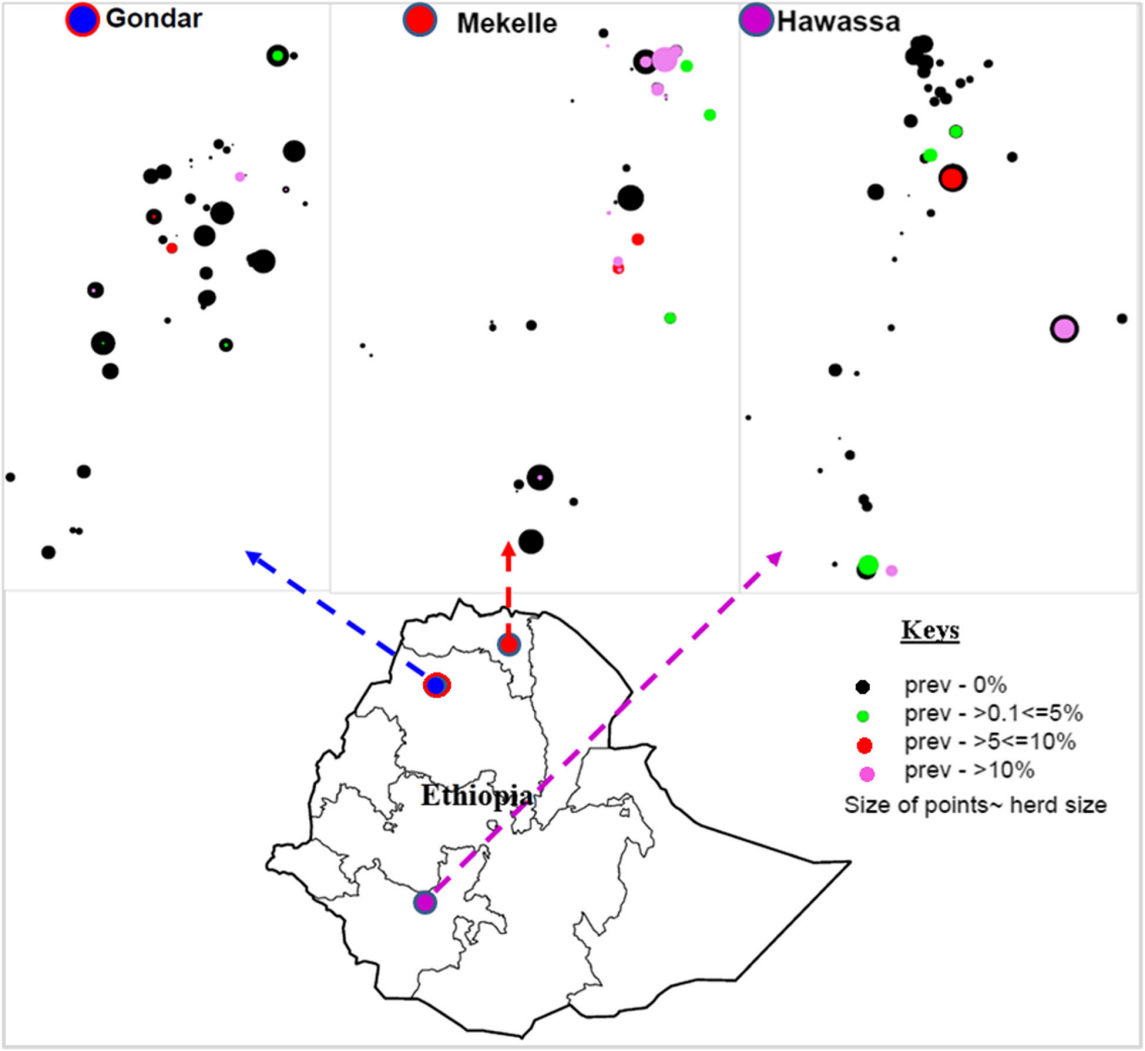
Geographic location of study sites and distributions of dairy farms in each site. Size of dots represents farm size while colors show BTB status: red indicates positive and black negative results recorded by tuberculin skin test. Base map source: http://maplibrary.org/library/stacks/Africa/Ethiopia/index.htm

The study was conducted in three selected cities of Ethiopian regional Governments namely Hawassa, Gondar, and Mekelle (Fig. 4). The cities were purposively selected due to the fact that the dairy industry has been rapidly growing in these areas in accordance with the Ethiopian Government long term plan to expand the dairy industry to achieve the need of animal sourced nutrition in these areas [2]. Hawassa represents the southern, Gondar the northwestern and Mekelle the northern emerging dairy belts of the country with the number of herds (animals) of more than 200 (5200), 440 (4800) and 260 (2600), respectively. These cities are densely populated with a human population size of about 0.3 Million in each city [42]. Their respective distances from the capital, Addis Ababa, are 273, 738 and 783 km.

### Data collection

The study involved 278 farms in total, of which 67, 66 and 81 were located, respectively in Hawassa, Gondar and Mekelle while 64 were located in other sites and served as cattle sources. Researchers described the objective of the study to the respondents before forwarding any of the questions to ensure that the feeling and mood of participants was good. Data were collected using a pretested questionnaire addressing specific questions on dairy cattle movement including number of cattle, batch size, purpose and date of movement (September 2013 to August 2018). Information was collected from farm owners and/or farm managers. To optimize the memory of the respondents the researchers assessed the history of each animal through focused conversation with the respondent walking within the barn where cattle were kept. The interview was made in such a way that respondents would feel secured about all information provided. Data on tuberculin testing and other pertinent animal level data were collected in parallel with the questionnaire survey.

### Herd classification based on tuberculin test

Herds were classified as infected or non-infected to BTB based on Single Intradermal Comparative Cervical Tuberculin (SICCT) test. Herds were classified as infected when at least one animal was found positive in the herd. We followed standard interpretation as described in OIE [43], where we considered a skin reaction as positive if the increase in skin thickness at the bovine site was more than 4 mm greater than the reaction shown at the site of the avian injection measured after 72 h of injection. SICCT test is known to have high diagnostic specificity (99.98%) [44] but imperfect (and variable) sensitivity (75–95.5%) [45–47]). The SICCT test is considered more reliable as a herd level test rather than as an individual animal test - hence we examine how network characteristics relate to herd level risk where we have a relatively higher confidence in the ability of the test to classify infected and non-infected herds. However, this work acknowledges the possibility of misclassifying herds with low prevalence of BTB.

### The network topology and metrics

Aggregates of cattle movement data were used to construct a directed static network. Farms from which cattle were sourced from or to which cattle were destined to go, were considered as nodes and cattle movements between farms as links or edges. The overall network topology was checked for small world or scale free structures, as both do have important roles in determining the nature of epidemics [48, 49]). The definition of a small world network is one where the clustering coefficient is significantly higher and the average shortest path length lower than that computed from a random network of equivalent magnitude i.e. the same number of nodes and links as the real network [35]. Similarly the network is considered scale free when the degree distribution follows a power law [50].

Node centralities relevant as possible targets for disease control [15, 51] were calculated by the indegree, outdegree, closeness, betweenness and eigenvectors. The indegree and outdegree centralities refer the number of incoming and outgoing cattle moves, respectively. Betweenness measures the frequency with which a farm is located on the shortest path length between pairs of other farms; and eigenvector centrality measures the degree to which a farm is connected to other well connected farms. The degree distribution was assessed following the guidelines described by Clauset et al. [52]. The network topology was described by using various network level metrics, including network diameter, average shortest path length, density, assortativity, clustering coefficients, modularity and network centralization. Node and network level metrics considered for the analyses were adapted as defined in Motta et al. [41], Dubé et al. [53] and Pavlopoulos et al. [54]. Definition of various node and network level terminologies are presented in Additional file 3: Table S3.

Key actors in the context of cattle movement networks refers the most important farm(s) in the network that have significant role in the functionality of the network, removing of which would result in the least possible cohesion of the network [55, 56]. Identification of such important farms was made based on a correlation analysis between node centrality measures. Centrality measures with weak correlations were considered to detect important farms in the network for they would show very low or none linear relation between them. The analytic approach followed the study conducted by Motta et al. [41] who used the method to identify key markets on a trade network.

### Network cohesiveness and reliability analysis

The overall network connectivity and structural features of the network were explored by conducting cohesive sub-group analyses based on k-core decomposition. A k-core is a sub-group in which each node is adjacent to at least a minimum number, k, of the other nodes in the sub-group. K-core decomposition allows identifying the core and periphery of the network**.** The largest connected components within the network, namely the giant strongly connected component (GSCC) and giant weakly connected component (GWCC) of the network were identified. The GSCC is the sub-group of nodes in which a node could be reached from every other node considering the directionality of links, whereas the GWCC is the sub-group of nodes for which directionality of the connections was disregarded. Further subsets of networks within the giant connected components that were more connected to each other than to the rest of the network were also identified using a greedy optimization community detection algorithm**.**

Vulnerability of the cohesiveness of the network structure due to targeted removal of farms was assessed using percolation analysis. This analysis examines the impact of progressively removing farms one after the other in the descending order of a given centrality measure on the structure of the network. Centrality measures utilized for this analysis involved indegree, outdegree, betweenness, closeness and eigenvector. The cohesiveness of the cattle movement network was evaluated by computing at each removal step on the size of the GWCC and size of the biggest community present in the remaining networks.

### Statistical analysis and graphics

Apparent prevalence was calculated using proportions of positive farms or animals from the total number of tested farms or cattle. Analyses of herd level risk factors were analyzed using generalized linear models (GLM) with binomial family and logit link. Variable were selected for the full model if the *p* value was less than 0.25 in the univariate model. Software used for the statistical analysis and graphics was R statistical software (version 3.4.1) (R Core Team) with R Studio editor using igraph [57], network [58], RColorBrewer [59], ggplot2 [60], poweRlaw [61], raster [62], glm2 [63], questionr [64], car [65], resourceSelection [66] and pROC [67] packages. In all cases, 95% confidence level and significance level of 5% were used to determine statistical significance.

### Data management and quality control

Data collection and tuberculin testing were carried out by trained and experienced personnel to avoid possible errors. The researcher closely supervised each step of data collection and ensured that data were collected properly. Completed questionnaires and animal level data collection formats were checked for completeness and presence of outliers on daily basis for prompt correction. Data were double entered and managed by a trained staff using the OpenClinica database (open source software, version 3.1; www.OpenClinica.com) and any entry errors were detected by the internal quality control system of the software.

## Additional files

Additional file 1: **Table S1.** Nodes and connections in the overall cattle movement network. (DOCX 14 kb)

Additional file 2: **Table S2.** Correlation between node centrality measures. (DOCX 14 kb)

Additional file 3: **Table S3.** Node and network level metrics definitions. (DOCX 16 kb)

Additional file 4: **Figure S1.** Key-actor analysis on the cattle movement network. Key-actor analysis for the full-network (D) and specific sites (regions) (A, B & C) based on correlation between betweenness and eigenvector centralities. Size and color-fade of the labels is relative to the value of residuals obtained through linear regression showing the deviation from a linear relationship. *Definition:* farms placed in quadrant (a) were farms which did not have any particular role in the network; (b) are pulse-takers; (c) are farms which tends to have both gate-keeper and pulse taker abilities; and (d) were gate-keepers. (TIF 466 kb)

Additional file 5: **Figure S2.** Core decomposition plot. Four cores identified with size of 53, 126, 89 and 9, respectively. (TIF 297 kb)

Additional file 6: **Figure S3.** Communities and vertexes by study sites (regions), as detected by greedy optimization algorism. Three bigger groups of farms for three loosely connected regions clustered together forming sub-networks generated after reducing vertices having no connections. Group of communities encompassing vertexes of similar color correspond to one region. Vertexes with dark-orange were based in Gondar; vertex color aquamarine corresponds to Mekelle; deep-sky-blue corresponds to Hawassa; yellow corresponds to farms which do not belong to any of the regions (these are ‘unknown’ with respect to detailed data). Communities were shown with various shades of colors to differentiate one from the other. (TIF 682 kb)

Additional file 7: **Figure S4.** Graphs showing relationship of community size and number of infected farms (bottom) and infected animals (upper). (TIF 275 kb)

## Data Availability

All data generated and /or analyzed during the current study are not publicly available but are available from the corresponding author on reasonable request.

## Abbreviations

BTB: Bovine tuberculosis
CC: Clustering coefficient
CI: Confidence interval
CSA: Central statistics agency
GLM: Generalized linear model
GSCC: Giant strong connected component
GWCC: Giant weak connected component
HF: Holstein friesian
LMP: Livestock master plan
OIE: Office for international animal health
OR: Odds ratio
r: Correlation coefficient
R^2^: Variance
UK: United Kingdom

## References

[1] CSA (2017). Report on livestock and livestock characteristics. Federal Democratic Republic of Ethiopia. Agricultural sample survey: volume II.

[2] Shapiro BI, Gebru G, Desta S, Negassa A, Nigussie K, Aboset G (2015). Ethiopia livestock master plan. ILRI Project Report.

[3] Tegegne A, Gebremedhin B, Hoekstra D, Belay B, Mekasha Y (2013). Smallholder dairy production and marketing systems in Ethiopia IPMS experiences and opportunities for market-oriented development. Working Paper No. 31.

[4] Ahmed MM, Ehui S, Assefa Y (2003). Dairy development in Ethiopia. Socio-economics and policy research working paper 58.

[5] Ameni G, Bonne P, Tibbo M (2003). A cross-sectional study of bovine Tuberculosis in selected dairy farms in Ethiopia. Int J App Res Vet Med.

[6] Ameni G, Aseffa A, Engers H, Young D, Gordon S, Hewinson G, Vordermeier M (2007). High prevalence and increased severity of pathology of bovine tuberculosis in Holsteins compared to zebu breeds under field cattle husbandry in Central Ethiopia. Clin Vaccine Immunol.

[7] Elias K, Hussein D, Gebeyehu M (2008). Status of bovine tuberculosis in Addis Ababa dairy farms. Rev Sci Tech Off Int Epiz.

[8] Tsegaye W, Aseffa A, Mache A, Mengistu Y, Berg S, Ameni G (2010). Conventional and molecular epidemiology of bovine Tuberculosis in dairy farms in Addis Ababa City, the Capital of Ethiopia. J Appl Res Vet Med.

[9] Firdessa R, Tschopp R, Wubete A, Sombo M, Hailu E, Erenso G (2012). High prevalence of bovine tuberculosis in dairy cattle in central ethiopia: implications for the dairy industry and public health. PloS One.

[10] Sibhat B, Asmare K, Demissie K, Ayelet G, Mamo G, Ameni G (2017). Bovine tuberculosis in Ethiopia: a systematic review and meta-analysis. Prev Vet Med.

[11] Kao RR, Danon L, Green DM, Kiss IZ (2006). Demographic structure and pathogen dynamics on the network of livestock movements in Great Britain. Proc R Soc Lond B Biol Sci.

[12] Ortiz-Pelaez A, Pfeiffer DU, Soares-Magalha RJ, Guitian FJ (2006). Use of social network analysis to characterize the pattern of animal movements in the initial phases of the 2001 foot and mouth disease (FMD) epidemic in the UK. Prev Vet Med.

[13] Robinson SE, Christley RM (2007). Exploring the role of auction markets in cattle movements within Great Britain. Prev Vet Med..

[14] Dubé C, Ribble C, Kelton D (2010). An analysis of the movement of dairy cattle through 2 large livestock markets in the province of Ontario, Canaeda. Can Vet J.

[15] Dubé C, Ribble C, Kelton D, Mcnab B (2009). A review of network analysis terminology and its application to foot-and-mouth disease modelling and policy development. Transbound Emerg Dis.

[16] Volkova VV, Howey R, Savill NJ, Woolhouse MEJ (2010). Sheep movement networks and the transmission of infectious diseases. PLoS One.

[17] Smith RP, Cook AJC, Christley RM (2013). Descriptive and social network analysis of pig transport data recorded by quality assured pig farms in the UK. Prev Vet Med.

[18] Brooks-Pollock E, Roberts GO, Keeling MJ (2014). A dynamic model of bovine tuberculosis spread and control in Great Britain. Nature.

[19] Gilbert M, Mitchell A, Bourn D, Mawdsley J, Clifton-Hadley R, Wint W (2005). Cattle movements and bovine tuberculosis in Great Britain. Nature.

[20] Craft Meggan E. (2015). Infectious disease transmission and contact networks in wildlife and livestock. Philosophical Transactions of the Royal Society B: Biological Sciences.

[21] Kenah E, Robins JM (2007). Second look at the spread of epidemics on networks. Phys Rev.

[22] Danon Leon, Ford Ashley P., House Thomas, Jewell Chris P., Keeling Matt J., Roberts Gareth O., Ross Joshua V., Vernon Matthew C. (2011). Networks and the Epidemiology of Infectious Disease. Interdisciplinary Perspectives on Infectious Diseases.

[23] Lentz Hartmut H. K., Koher Andreas, Hövel Philipp, Gethmann Jörn, Sauter-Louis Carola, Selhorst Thomas, Conraths Franz J. (2016). Disease Spread through Animal Movements: A Static and Temporal Network Analysis of Pig Trade in Germany. PLOS ONE.

[24] Mohr S, Deason M, Churakov M, Doherty T, Kao RR (2018). Manipulation of contact network structure and the impact on foot-and-mouth disease transmission. Prev Vet Med.

[25] OIE. Terrestrial Animal Health Code: Bovine Tuberculosis Complex. 2015. Available from: http://www.oie.int/index.php?id=169&L=0&htmfile=chapitre_bovine_tuberculosis.htm

[26] Erdos P, Renyi A (1960). On the evolution of random graphs. Publ Math Inst Hungar Acad Sci.

[27] Kiss IZ, Green DM, Kao RR (2006). The network of sheep movements within Great Britain: network properties and their implications for infectious disease spread. J R Soc Interface.

[28] Corner LA, Pfeiffer DU, Morris RS (2003). Social-network analysis of Mycobacterium bovis transmission among captive brushtail possums (Trichosurus vulpecula). Prev Vet Med.

[29] Mekonnen GA, Conlan AJK, Berg S, Ayele BT, Alemu A, Guta S (2019). Prevalence of bovine tuberculosis and its associated risk factors in the emerging dairy belts of regional cities in Ethiopia. Prev Vet Med.

[30] Caminiti A, Pelone F, La Torre G, De Giusti M, Saulle R, Mannocci A (2016). Control and eradication of tuberculosis in cattle: a systematic review of economic evidence. Vet Rec.

[31] Cousins DV (2001). Mycobacterium bovis infection and control in domestic livestock. Rev Sci Tech.

[32] Reilly LA, Courtenay O (2007). Husbandry practices, badger sett density and habitat composition as risk factors for transient and persistent bovine tuberculosis on UK cattle farms. Prev Vet Med.

[33] Baptista FM, Nunes T, Almeida V, Louzã A (2008). Cattle movements in Portugal – an insight into the potential use of network analysis. Rev Port Cardiol Ciec Vet.

[34] Eames KTD, Keeling MJ (2003). Contact tracing and disease control. Proc R Soc Lond B.

[35] Watts DJ, Strogatz SH (1998). Collective dynamics of ‘small-world’ networks. Nature.

[36] Keeling MJ, Eames KTD (2005). Networks and epidemic models. J R Soc Interface.

[37] Riley S, Fraser C, Donnelly CA, Ghani AC, Abu-Raddad LJ, Hedley AJ (2003). Transmission dynamics of the etiological agent of SARS in Hong Kong: impact of public health interventions. Science.

[38] Hufnagel L, Brockmann D, Geisel T (2004). Forecast and control of epidemics in a globalized world. Proc Natl Acad Sci U S A.

[39] Kiss IZ, Green DM, Kao RR (2006). Infectious disease control using contact tracing in random and scale-free networks. J R Soc Interface.

[40] Pautasso M, Jeger MJ (2008). Epidemic threshold and network structure: the interplay of probability of transmission and of persistence in small-size directed networks. Ecol Complex.

[41] Motta P, Porphyre T, Handel I, Hamman SM, Ngwa VN, Tanya V (2017). Implications of the cattle trade network in Cameroon for regional disease prevention and control. Sci Rep.

[42] CSA (2015). Central Statistical Agency. Federal Democratic Republic of Ethiopia (internet).

[43] OIE (2009). Terrestrial Manual: Bovine Tuberculosis.

[44] Goodchild AV, Downs SH, Upton P, Wood JLN, de La Rua-Domenech R. Specificity of the comparative skin test for bovine tuberculosis in Great Britain. Vet Rec. 2015;177:258 Available from: 10.1136/vr.10296 PMC4602248 26338518

[45] Karolemeas Katerina, de la Rua-Domenech Ricardo, Cooper Roderick, Goodchild Anthony V., Clifton-Hadley Richard S., Conlan Andrew J. K., Mitchell Andrew P., Hewinson R. Glyn, Donnelly Christl A., Wood James L. N., McKinley Trevelyan J. (2012). Estimation of the Relative Sensitivity of the Comparative Tuberculin Skin Test in Tuberculous Cattle Herds Subjected to Depopulation. PLoS ONE.

[46] de la Rua-Domenech R, Goodchild AT, Vordermeier HM, Hewinson RG, Christiansen KH, Clifton-Hadley RS (2006). Ante mortem diagnosis of tuberculosis in cattle: a review of the tuberculin tests, γ-interferon assay and other ancillary diagnostic techniques. Res Vet Sci.

[47] Monaghan ML, Doherty ML, Collins JD, Kazda JF, Quinn PJ (1994). The tuberculin test. Vet Microbiol.

[48] Barabási AL, Bonabeau E (2003). Scale-free networks. Sci Am.

[49] Liu Meng, Li Daqing, Qin Pengju, Liu Chaoran, Wang Huijuan, Wang Feilong (2015). Epidemics in Interconnected Small-World Networks. PLOS ONE.

[50] Wang XF, Chen G (2003). Complex networks small-world, scale-free and beyond. IEEE Circuits Syst Mag.

[51] Martínez-López B, Perez AM, Sánchez-Vizcaíno JM (2009). Social network analysis. Review of general concepts and use in preventive veterinary medicine. Transbound Emerg Dis.

[52] Clauset A, Shalizi CR, Newman MEJ (2009). Power-law distributions in empirical data. SIAM Rev.

[53] Dubé C, Ribble C, Kelton D, Mcnab B (2011). Introduction to network analysis and its implications for animal disease modelling. Rev Sci Tech Off Int Epiz.

[54] Pavlopoulos GA, Secrier M, Moschopoulos CN, Soldatos TG, Kossida S, Aerts J (2011). Using graph theory to analyze biological networks. BioData Min.

[55] Borgatti SP (2006). Identifying sets of key players in a social network. Comput Math Organ Theory.

[56] Conway D (2010). Analyzing terrorist networks - theories and techniques.

[57] Csardi G, Nepusz T. The igraph software package for complex network research. Inter J Complex Syst. 2006;1695:1–9. Available from: http://igraph.org

[58] Butts C. network: classes for relational data. The Statnet project. R package version 1.13.0.1; 2015. Available from: https://cran.r-project.org/web/packages/network/index.html

[59] Neuwirth E (2014). ColorBrewer: color brewer palettes. R package version 1.1–2.

[60] Wickham H, Chang W (2016). ggplot2: create elegant data visualisations using the grammar of graphics. R package version 2.2.1.

[61] Gillespie CS (2015). Fitting heavy tailed distributions: the power-law package. J Stat Softw.

[62] Hijmans RJ, van Etten J, Joe Cheng J, Sumner M, Mattiuzzi M, Greenberg JA, et al. raster: geographic data analysis and modeling. R package version 2.8–4;2018. Available from: https://cran.r-project.org/web/packages/raster/index.html

[63] Marschner Ian,C. (2011). glm2: Fitting Generalized Linear Models with Convergence Problems. The R Journal.

[64] Barnier J, Briatte F, Larmarange J. questionr: functions to make surveys processing easier. R package version 0.6.2;2017. Available from: https://cran.r-project.org/web/packages/questionr/index.html

[65] Fox J, Weisberg S, Adler D, Bates D, Baud-Bovy G, Ellison S, et al. car: companion to applied regression. R package version 2.1–5;2017. Available from: https://cran.r-project.org/web/packages/car/index.html.

[66] Lele SR, Keim JL, Solymos P. ResourceSelection: resource selection (probability) functions for use-availability data. R package version 0.3-2;2017. Available from: https://cran.r-project.org/web/packages/ResourceSelection/index.html

[67] Robin X, Turck N, Hainard A, Tiberti N, Lisacek F, Sanchez JC. pROC: an open-source package for R and S+ to analyze and compare ROC curves. BMC Bioinformatics. 2011;12:77. Available from: 10.1186/1471-2105-12-77 PMC3068975 21414208

