# Supplementary material for: Network analysis of dairy cattle movement and associations with bovine tuberculosis spread and control in emerging dairy belts of Ethiopia
Source: BMC Vet Res. 2019 Jul 26;15:262. doi: 10.1186/s12917-019-1962-1 (PMC6660945; doi:10.1186/s12917-019-1962-1)
Supplement: Supplementary file 1 — Table S1. Nodes and connections in the overall cattle movement network. (DOCX 14 kb) [file 12917_2019_1962_MOESM1_ESM.docx]

**Additional file 1: Table S1:** Number of nodes and connections in the overall cattle movement network

| Number connection per node (degree) | Nodes | |  | Connections | |
| --- | --- | --- | --- | --- | --- |
|  | Number of nodes | % connected |  | Number of connections | % connection |
| 0 | 53 | 19 |  | 0 | 0 |
| ≥1 and <5 | 190 | 68 |  | 324 | 55.5 |
| ≥5 | 35 | 13 |  | 260 | 44.5 |
| Total | 278 | 100 |  | 584 | 100 |
