# Supplementary material for: Network analysis of dairy cattle movement and associations with bovine tuberculosis spread and control in emerging dairy belts of Ethiopia
Source: BMC Vet Res. 2019 Jul 26;15:262. doi: 10.1186/s12917-019-1962-1 (PMC6660945; doi:10.1186/s12917-019-1962-1)
Supplement: Supplementary file 2 — Table S2. Correlation between node centrality measures. (DOCX 14 kb) [file 12917_2019_1962_MOESM2_ESM.docx]

**Additional file 2: Table S2:** Correlation between centralities (Method=Spearman)

|  | Indegree | Outdegree | Closeness | Betweenness | Eigenvector |
| --- | --- | --- | --- | --- | --- |
| Indegree | 1 |  |  |  |  |
| Outdegree | -0.25 | 1 |  |  |  |
| Closeness | 0.40 | 0.49 | 1 |  |  |
| Betweenness | 0.52 | 0.34 | 0.34 | 1 |  |
| Eigenvector | 0.33 | 0.48 | 0.83 | 0.24 | 1 |
