# Supplementary material for: Network analysis of dairy cattle movement and associations with bovine tuberculosis spread and control in emerging dairy belts of Ethiopia
Source: BMC Vet Res. 2019 Jul 26;15:262. doi: 10.1186/s12917-019-1962-1 (PMC6660945; doi:10.1186/s12917-019-1962-1)
Supplement: Supplementary file 3 — Table S3. Node and network level metrics definitions. (DOCX 16 kb) [file 12917_2019_1962_MOESM3_ESM.docx]

**Additional file 3: Table S3:** Node and network level metrics

(Definition adapted from Motta *et al.,* 2017; Dubé *et al.,* 2011; Pavlopoulos *et al.,* 2011)

| **Term** | **Definition** |
| --- | --- |
| **Node-level metrics** | |
| Indegree | Computed accounting for both the number of connections that each node receives in a defined period and the weights of these connections, hence a measure of the potential sources or origins of infection in that range of time |
| Outdegree | Computed accounting for both the number of connections that each node sends in a defined period and the weights of these connections, and therefore the number of potential destinations of infection in that range of time. |
| Closeness | The mathematical inverse of farness or the inverse of the average distance between a node and all the other nodes in a network |
| Betweenness | Frequency with which a node is located on the shortest path length between any pairs of nodes, accounting that the connection between nodes might be stronger along paths with more intermediate nodes that are strongly connected than paths with fewer weakly-connected links. In other words, it is a measure of the tendency of connecting nodes which would be otherwise disconnected. |
| Eigenvector | Measures the degree to which a node is connected to other well connected nodes. It takes advantage of a mathematical property of networks that allows for the easy calculation of how well connected a node is to other well connected nodes |
| **Network-level metrics** | |
| Density | Number of edges (C) in the observed network relative to the total number of possible edges (k(k-1) in a completely connected network, which is C / k(k-1) |
| Assortativity | Quantifies the tendency of individual nodes to connect with other nodes which  are similar to themselves in terms of a centrality measure, it shows the propensity of epidemiologically similar nodes to be connected to each other, |
| Modularity | the partitioning of the network into internally well-connected groups |
| Shortest average path length | The shortest average number of edges that must be traversed to connect any two nodes in the network, without accounting for the temporal dimension of the connections |
| Diameter | The shortest distance/path length between the two most distant connected nodes in the network (= the longest of all the calculated path lengths). |
| Clustering Coefficient (transitivity) | Proportion of pairs of neighbors of a given node which are connected or the fraction of triplets that form triangles. It measures that probability that adjacent nodes of a network are connected. In other words, if i is connected to j, and j is connected to k, what is the probability that i is also connected to k? |
| Giant strongly connected component (GSCC) | The largest subset of nodes that are mutually reachable through directed paths |
| Giant weakly connected component(GWCC) | The largest subset of nodes that are mutually reachable through undirected paths,  therefore not accounting for the directionality of connections |
| Key actors | They are most important nodes in the network that have significant role in the functionality of the network by serving as gate keeper’s and /or pulse taker’s function removing of which would result in the least possible cohesion of the network |
| Pulse taker | Nodes with higher eigenvector values and shortest paths to all other nodes having easy access to the other central markets and to the rest of the network. |
| Gate keeper | Nodes that have higher value of betweenness are considered as gate keepers due to their ability to bridge between the functional nodes of the network and wider community of nodes. |
