# Supplementary figures and images for: Network analysis of dairy cattle movement and associations with bovine tuberculosis spread and control in emerging dairy belts of Ethiopia
Source: BMC Vet Res. 2019 Jul 26;15:262. doi: 10.1186/s12917-019-1962-1 (PMC6660945; doi:10.1186/s12917-019-1962-1)

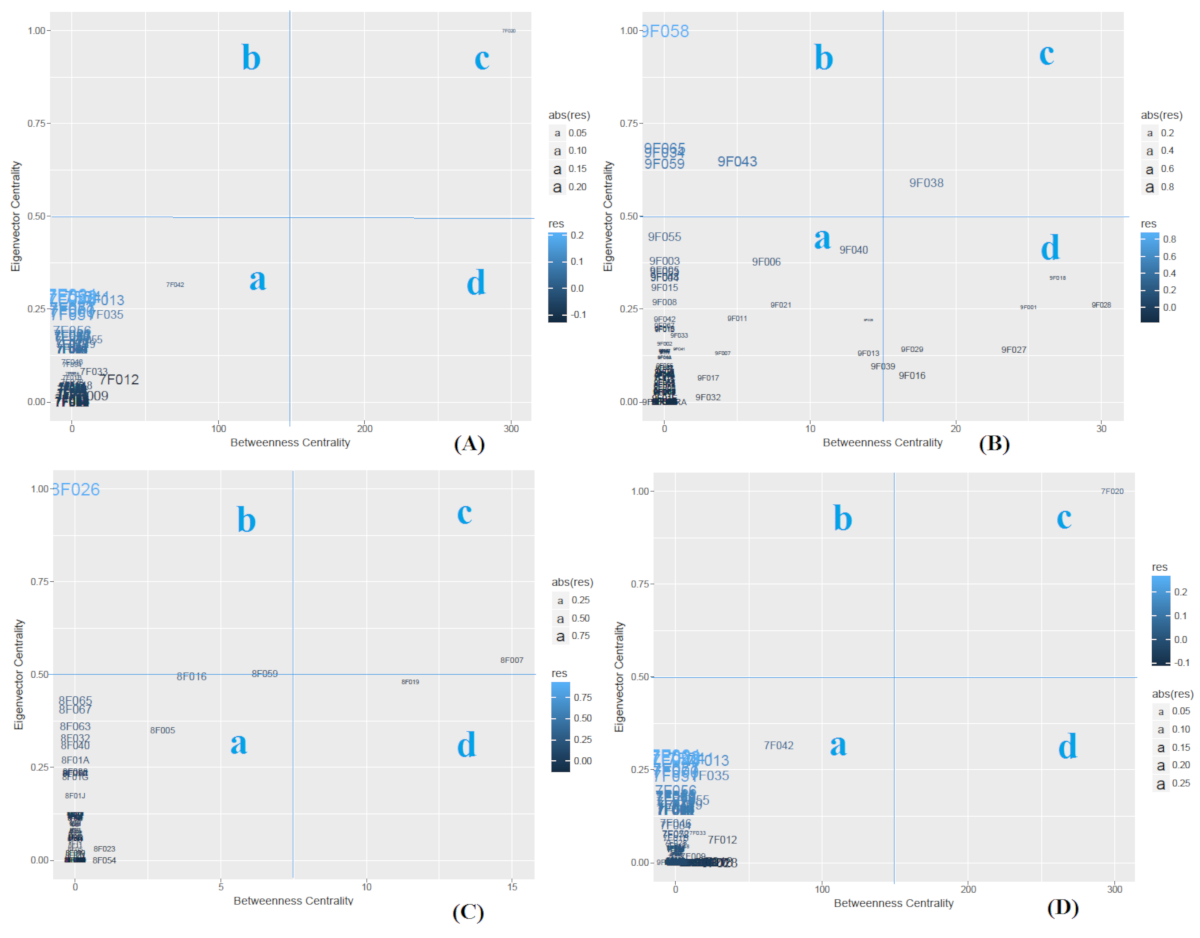

Supplement: Supplementary file 4 — Figure S1. Key-actor analysis on the cattle movement network. Key-actor analysis for the full-network (D) and specific sites (regions) (A, B & C) based on correlation between betweenness and eigenvector centralities. Size and color-fade of the labels is relative to the value of residuals obtained through linear regression showing the deviation from a linear relationship. Definition: farms placed in quadrant (a) were farms which did not have any particular role in the network; (b) are pulse-takers; (c) are farms which tends to have both gate-keeper and pulse taker abilities; and (d) were gate-keepers. (TIF 466 kb) [file 12917_2019_1962_MOESM4_ESM.tif]

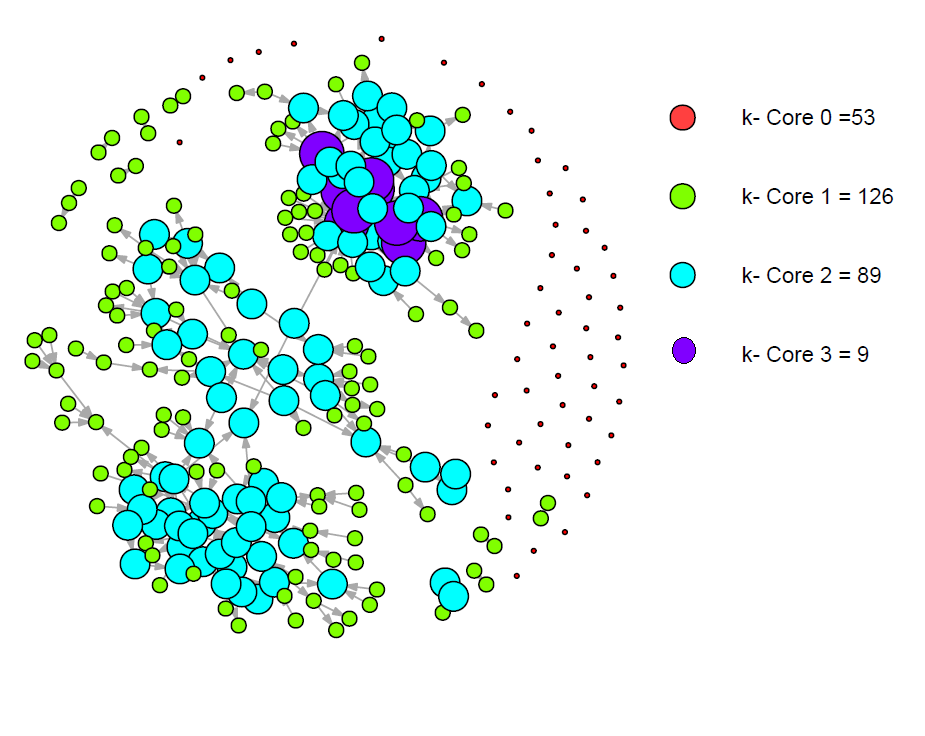

Supplement: Supplementary file 5 — Figure S2. Core decomposition plot. Four cores identified with size of 53, 126, 89 and 9, respectively. (TIF 297 kb) [file 12917_2019_1962_MOESM5_ESM.tif]

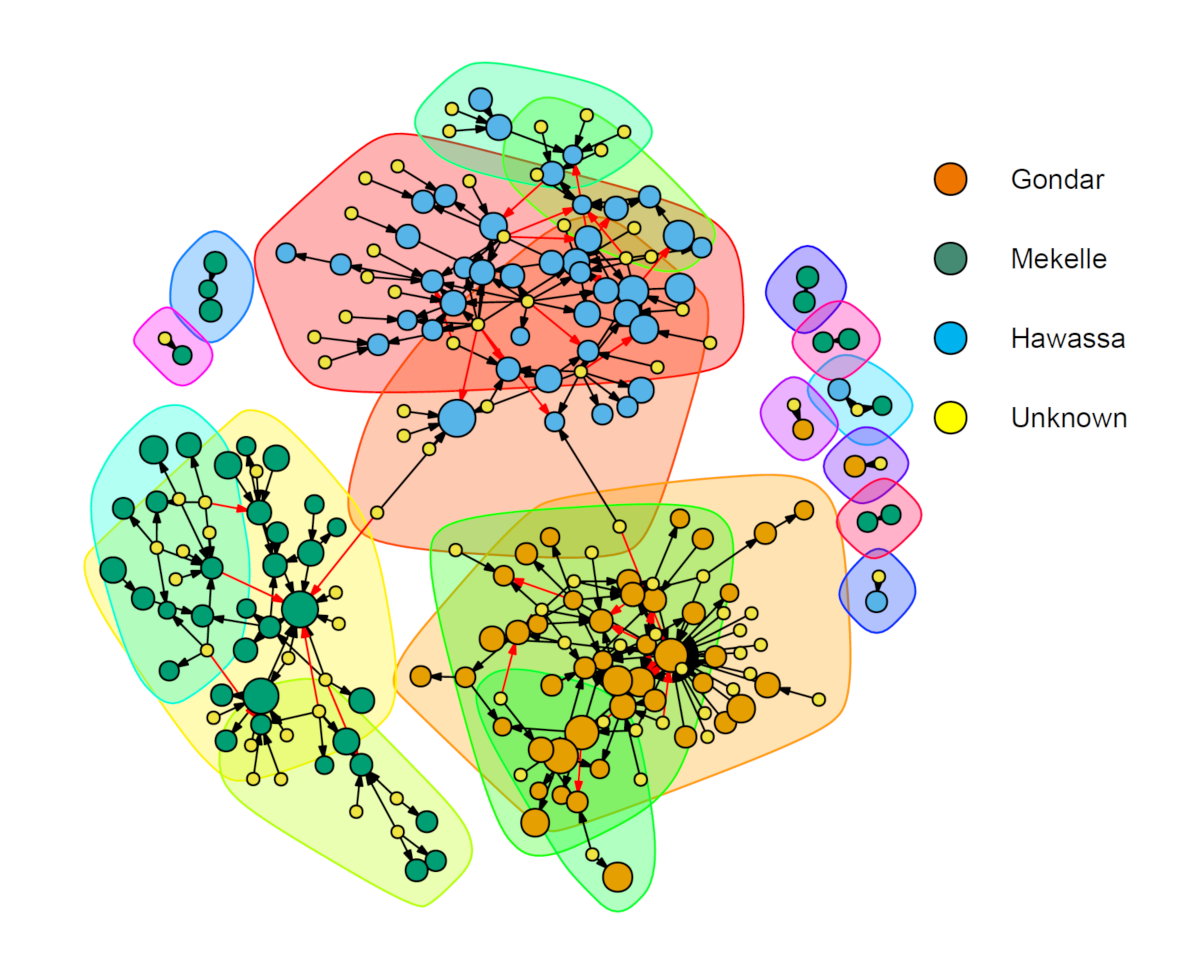

Supplement: Supplementary file 6 — Figure S3. Communities and vertexes by study sites (regions), as detected by greedy optimization algorism. Three bigger groups of farms for three loosely connected regions clustered together forming sub-networks generated after reducing vertices having no connections. Group of communities encompassing vertexes of similar color correspond to one region. Vertexes with dark-orange were based in Gondar; vertex color aquamarine corresponds to Mekelle; deep-sky-blue corresponds to Hawassa; yellow corresponds to farms which do not belong to any of the regions (these are ‘unknown’ with respect to detailed data). Communities were shown with various shades of colors to differentiate one from the other. (TIF 682 kb) [file 12917_2019_1962_MOESM6_ESM.tif]

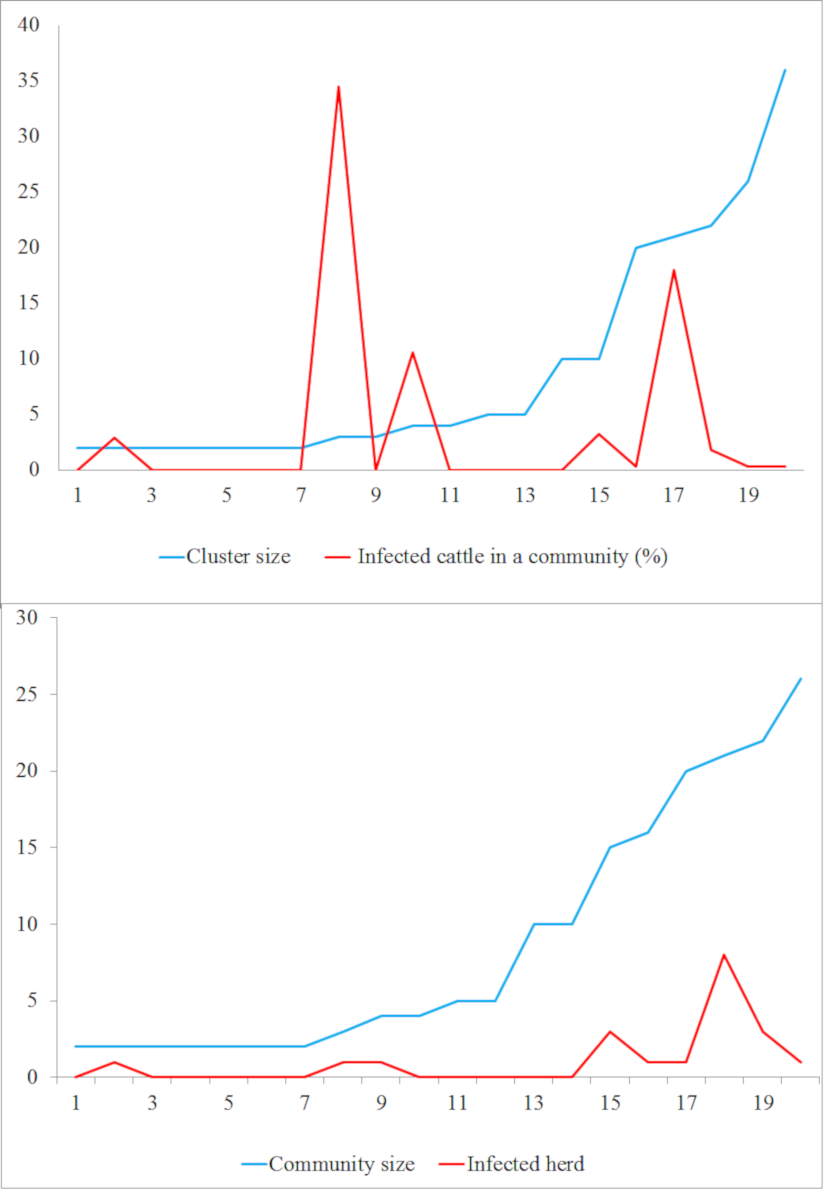

Supplement: Supplementary file 7 — Figure S4. Graphs showing relationship of community size and number of infected farms (bottom) and infected animals (upper). (TIF 275 kb) [file 12917_2019_1962_MOESM7_ESM.tif]
